# Suicide Attempt and Suicidal Drug Overdose in Chronic Obstructive Pulmonary Disease Patients With or Without Depression

**DOI:** 10.3389/fpsyt.2020.00270

**Published:** 2020-04-15

**Authors:** Chi-Yu Lin, Tomor Harnod, Cheng-Li Lin, Chia-Hung Kao

**Affiliations:** ^1^ Stroke Care Center and Department of Neurology, Yumin Hospital, Nantou, Taiwan; ^2^ Department of Neurosurgery, Hualien Tzu Chi Hospital, Buddhist Tzu Chi Medical Foundation, Hualien, Taiwan; ^3^ College of Medicine, Tzu Chi University, Hualien, Taiwan; ^4^ Management Office for Health Data, China Medical University Hospital, Taichung, Taiwan; ^5^ College of Medicine, China Medical University, Taichung, Taiwan; ^6^ Graduate Institute of Biomedical Sciences and School of Medicine, College of Medicine, China Medical University, Taichung, Taiwan; ^7^ Department of Nuclear Medicine and PET Center, China Medical University Hospital, Taichung, Taiwan; ^8^ Department of Bioinformatics and Medical Engineering, Asia University, Taichung, Taiwan; ^9^ Center of Augmented Intelligence in Healthcare, China Medical University Hospital, Taichung, Taiwan

**Keywords:** chronic obstructive pulmonary disease, depression, National Health Insurance, suicide, suicidal drug overdose

## Abstract

**Background:**

To determine differences in the incidence and risks of suicide attempt (SA) and suicidal drug overdose (SDO) between chronic obstructive pulmonary disease (COPD) patients with and without comorbid depression by using data from Taiwan’s National Health Insurance Research Database.

**Methods:**

We analyzed the data of patients aged ≥20 years who had received a COPD diagnosis between 2000 and 2012. These COPD patients were divided into those with and without depression, and they were compared against a cohort from the general population. We calculated adjusted hazard ratios and the corresponding 95% confidence intervals for SA and SDO in the three cohorts after adjustment for age, sex, and comorbidities.

**Results:**

Until the end of 2012, 5.81% of patients with COPD developed depression. The incidence of SA and SDO in COPD patients with and without depression was 29.7 and 4.69 per 10,000 person-years and 71.2 and 20.9 per 10,000 person-years, respectively. COPD patients with depression had 13.6- and 10.0-fold higher risks of SA and SDO, respectively, than did controls. Particularly, an increased risk of SA caused by the enhancement effects of depression on COPD was noted in patients aged less than 50 years.

**Conclusion:**

SA and SDO risks are extremely high in Taiwanese COPD patients with depression. Our findings suggest that clinicians should be aware that for COPD patients with comorbid depression, prescribing a large amount of medications may be associated with SA risk through SDO.

## Introduction

Globally, more than 800,000 people commit suicide every year, and the prevalence of completed suicide has progressively increased over the past decades (1). The annual prevalence of suicidal ideation and suicide attempt (SA) has been estimated to be 2.3% and 0.4% in Austria and 3.7% and 0.5% in the United States, respectively (2, 3). In European countries, the estimated rate of suicidal lethality is much higher in men than in women (13.9% vs. 4.1%) (4). The main possible reason for this sex difference is the different methods of SA adopted by men and women; violent methods are more common among men, and self-intoxication or suicidal drug overdose (SDO) is the most frequently chosen method among women (5). Therefore, we believe that adequately identifying individuals at a high risk of SA and interrupting their SA are urgently required for preventing suicide (6).

Depression is a well-established predictor of suicidal behaviors (7, 8). Researchers have investigated interactions between suicidality and medical conditions for years; however, only few researchers have conducted population-based studies on this topic (9, 10). In a recent large population-based cross-national study, Scott et al. reported several physical conditions that are potential independent risk factors for suicidality (10). Whether the probability of suicide is high in patients with chronic medical conditions or whether it is noted only in patients with specific disorders remains controversial (11). Sanna et al. reported an association between suicidality and several medical disorders; however, no association was noted between suicidality and pulmonary disease (12).

The Taiwanese government established the National Health Insurance (NHI) program in 1995, which covers approximately 99% of the residents of Taiwan for the past 2 decades (13). Both chronic obstructive pulmonary disease (COPD) and depression are common chronic disorders in the National Health Insurance Research Database (NHIRD). Furthermore, pneumonia and COPD were the third and seventh causes of death in 2018 in Taiwan. As COPD is one of the major chronic physical disorders among elderly people, we believe that SA and SDO risks should be different between the general population and COPD patients with and without comorbid depression. Investigating these differences might be beneficial for the medical care systems in both Taiwan and other East Asian countries due to similarities in their ethnicity and cultural heritage (14).

## Methods

### Data Source

The NHIRD of Taiwan is maintained by the National Health Research Institutes of Taiwan. It contains comprehensive NHI records of almost 99% of the population of Taiwan (13). The database includes the de-identified medical records of inpatients and outpatients and their prescription drug and other medical service records. Only patients with hospitalization records were included in this study. Diagnoses were defined based on International Classification of Diseases, Ninth Revision, Clinical Modification (ICD-9-CM) codes. The Research Ethics Committee of China Medical University and Hospital in Taiwan approved the study (CMUH104-REC2-115-CR4).

### Study Population

To assess the differences in SA and SDO risks between COPD patients with and without depression, 4 cohorts were defined in this study: the total COPD cohort comprising patients aged ≥20 years diagnosed with COPD (ICD-9-CM codes 491, 492, 494, and 496), the COPD with depression cohort comprising COPD patients with depression (ICD-9-CM codes 296.2, 296.3, 296.82, 300.4, and 311), the COPD without depression cohort comprising COPD patients without depression, and the comparison cohort comprising individuals without COPD and depression during 2000–2012. The index date was the date of COPD diagnosis. Controls (individuals without COPD and depression) were frequency matched based on sex, age, and index year at a ratio of 1:2. The primary outcome was the occurrence of SA (ICD-9-CM codes E950–E959) and SDO in NHIRD records. SDO was defined based on an emergency department visit, a clinic visit, or hospitalization with the ICD-9-CM codes 960-979 but without E codes. The study excluded patients aged <20 years and patients with SA or SDO before the index date. The same exclusion criteria were applied for the comparison cohort.

Comorbidities comprised several mental-health-associated diseases such as schizophrenia (ICD-9-CM code 295), alcohol-related illness (ICD-9-CM codes 291, 303, 305.00, 305.01, 305.02, 305.03, 571.0, 571.1, 571.3, 790.3, and V11.3), anxiety (ICD-9-CM code 300.00), mental disorders (ICD-9-CM codes 290-319), and insomnia (ICD-9-CM codes 307.4 and 780.5), and these comorbidities with at least one hospitalization record before the index date were included in the analysis. The end date of the follow-up period was the date of SA or SDO occurrence, patient death, patient withdrawal from the NHI program, or December 31, 2012, whichever occurred first.

### Statistical Analyses

Categorical and continuous variables in this study are presented by number (%) and mean ± standard deviation, respectively. The chi-square test for categorical variables and analysis of variance for continuous variables were performed to examine the differences in these variables among the cohorts. Incidence rates of SA and SDO were calculated by dividing SA and SDO events by person-years (every 10,000 person-years). Cumulative incidence curves of SA and SDO in the cohorts were plotted using the Kaplan–Meier method, and log-rank tests were applied to test the differences in the curves. Hazard ratios (HRs), adjusted HRs (aHRs), and 95% confidence intervals (95% CIs) of SA and SDO were calculated using Cox proportional hazard models to evaluate the risks of SA and SDO among the cohorts. The level of significance was set at.05. All data were analyzed using SAS 9.4 software (SAS Institute Inc., Cary, NC, USA).

## Results

Four cohorts were defined in this study: total COPD (n = 361,703), COPD without depression (n = 340,694), COPD with depression (n = 21,009), and comparison (n = 721,550; without COPD and depression) cohorts. Because sex, age, and index year were frequency matched, no significant differences were found in sex (*P* =.39) and age (*P* =.78) among the cohorts. Significant differences were observed in monthly income, urbanization level, occupation category, and comorbidities among these cohorts (*P* < .001; **Table 1**).

**Table 1:** Distribution of demographic characteristics and comorbidities among COPD cohorts and the comparison cohort.

	Total COPD (N=361,703)		COPD without depression (N=340,694)		COPD with depression (N=21,009)		Comparison cohort (N=721,550)		p-value^a^
	n	%	n	%	n	%	n	%	
**Sex**									0.39
Women	232,082	32.2	108,264	31.8	7,779	37.0	116,043	32.1	
Men	489,468	67.8	232,430	68.2	13,230	63.0	245,660	67.9	
**Age stratified**									0.78
≤49	54,800	7.59	25,334	7.44	2,066	9.83	27,400	7.58	
50–64	118,596	16.4	55,627	16.3	3,671	17.5	59,298	16.4	
≥65	548,154	76.0	259,733	76.2	15,272	72.7	275,005	76.0	
Age, mean ± SD^§^	72.3 ± 13.6		72.4 ± 13.6		70.9 ± 13.9		71.3 ± 13.6		<0.001
**Monthly income** ^†^									<0.001
<15,000	266,408	36.9	128,651	37.8	9,781	46.6	138,432	38.3	
15,000−19,999	310,350	43.0	166,514	48.9	8,738	41.6	175,252	48.5	
≥20,000	144,792	20.1	45,529	13.4	2,490	11.9	48,019	13.3	
**Urbanization level** ^‡^									<0.001
1 (highest)	192,102	26.6	67,221	19.7	4,334	20.6	71,555	19.8	
2	197,342	27.4	90,418	26.5	6,160	29.3	96,578	26.7	
3	117,049	16.2	57,617	16.9	3,198	15.2	60,815	16.8	
4 (lowest)	215,057	29.8	125,438	36.8	7,317	34.8	132,755	36.7	
**Occupation category** ^&^									<0.001
Office worker	303,590	42.1	112,169	32.9	6,349	30.2	118,518	32.8	
Laborer	294,313	40.8	162,148	47.6	8,416	40.1	170,564	47.2	
Other	123,647	17.1	66,377	19.5	6,244	29.7	72,621	20.1	
**Comorbidity**									
Schizophrenic	1,835	0.25	2,696	0.79	47	2.26	3,170	0.88	<0.001
Alcohol-related illness	3,416	0.47	8,400	2.47	1,582	7.53	9,982	2.76	<0.001
Anxiety	2,918	0.40	5,740	1.68	2,283	10.9	8,023	2.22	<0.001
Mental disorders	16,367	2.27	33,533	9.84	–	–	33,533	9.27	<0.001
Insomnia	7,350	1.02	15,367	4.51	4,010	19.1	19,377	5.36	<0.001

^a^COPD, chronic obstructive pulmonary disease; Chi-square test; ^§^ANOVA, analysis of variance; SD, standard deviation. ^†^New Taiwan Dollar (NTD), 1 NTD is equal to 0.03 USD. ^‡^The urbanization level was divided into 4 levels based on the population density of the residential area, where level 1 was the most urbanized and level 4 was the least urbanized. ^&^Other occupation categories included those who were primarily retired, unemployed, and low-income people.

**Figure 1:**
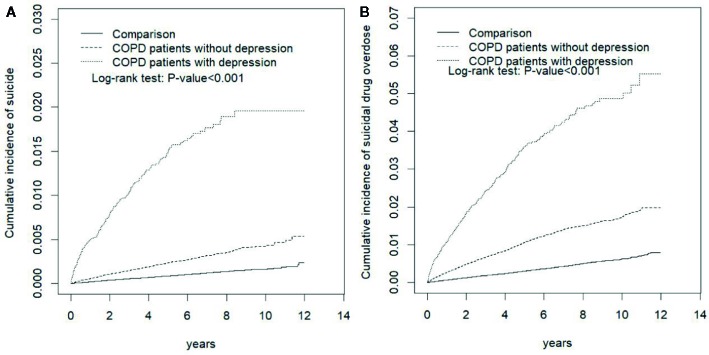
Comparison of the Cumulative Incidence of **(A)** Suicide Attempt and **(B)** Suicidal Drug Overdose Among COPD Patients With Depression, COPD Patients Without Depression, and the Normal Population. COPD, chronic obstructive pulmonary disease.

**Figure 1** displays the cumulative incidence of SA and SDO. The figure indicates significant differences in the cumulative incidence rates of SA and SDO among the COPD with and without depression cohorts and the comparison cohort. **Table 2** presents the incidence rates of and HRs of SA across different demographic characteristics. Compared with the reference group for each variable, a significantly high SA risk was observed after adjustment for demographic characteristics and comorbidities in COPD patients (aHR, 3.06; 95% CI, 2.72–3.45), COPD patients without depression (aHR, 2.49; 95% CI, 2.19–2.82), and COPD patients with depression (aHR, 13.6; 95% CI, 11.3-16.4) as well as in patients aged <50 years (aHR, 1.37; 95% CI, 1.11–1.69), with monthly incomes of New Taiwan Dollar (NTD) < 15,000 (aHR, 1.50; 95% CI, 1.19–1.87) and NTD15,000–19,999 (aHR, 1.48, 95% CI, 1.19–1.83), living in a residential area with a low urbanization level (level 2: aHR, 1.36; 95% CI, 1.14–1.62; level 3: aHR, 1.32; 95% CI, 1.08–1.61; and level 4: aHR, 1.33; 95% CI, 1.11–1.59), who worked as laborers (aHR, 1.37; 95% CI, 1.16–1.62), with alcohol-related illness (aHR, 2.14; 95% CI, 1.64–2.79), and with anxiety (aHR, 1.38; 95% CI, 1.02–1.87) (**Table 2**).

**Table 2:** Incidence and risk factors for suicide attempt across different factors.

Variable	Event	PY	Rate^#^	Crude HR (95% CI)	Adjusted HR^$^ (95% CI)
**COPD**					
None	508	300,7849	1.69	1.00	1.00
All	712	1,156,091	6.16	3.58(3.20, 4.02)***	3.06(2.72, 3.45)***
COPD without depression	511	1,088,444	4.69	2.73(2.41, 3.09)***	2.49(2.19, 2.82)***
COPD with depression	201	67,467	29.7	17.3(14.7, 20.4)***	13.6(11.3, 16.4)***
**Age group, years**					
≤49	153	399,532	3.83	1.52(1.23, 1.87)***	1.37(1.11, 1.69)**
50-64	202	792,705	2.55	1.00	1.00
≥65	865	2,971,704	2.91	1.11(0.96, 1.30)	1.14(0.97, 1.34)
**Sex**					
Women	372	1,384,182	2.69	1.00	1.00
Men	848	2,779,758	3.05	1.13(1.00, 1.28)	
**Monthly income** ^†^					
<15,000	433	1,535,721	2.82	1.69(1.39, 2.06)***	1.50(1.19, 1.87)***
15,000−19,999	660	1,862,091	3.54	2.13(1.76, 2.58)***	1.48(1.19, 1.83)***
≥20,000	127	766,128	1.66	1.00	1.00
**Urbanization level** ^‡^					
1 (highest)	198	1,023,850	1.93	1.00	1.00
2	350	1,139,992	3.07	1.59(1.33, 1.89)***	1.36(1.14, 1.62)***
3	205	680,968	3.01	1.56(1.28, 1.89)***	1.32(1.08, 1.61)***
4 (lowest)	467	1,319,131	3.54	1.83(1.55, 2.16)***	1.33(1.11, 1.59)***
**Occupation category** ^&^					
Office worker	346	1,649,829	2.10	1.00	1.00
Laborer	644	1,772,567	3.63	1.73(1.52, 1.97)***	1.37(1.16, 1.62)***
Other	230	741,545	3.10	1.47(1.25, 1.74)***	1.07(0.88, 1.29)
**Comorbidity**					
Schizophrenia					
No	1205	4,147,187	2.91	1.00	1.00
Yes	15	16,754	8.95	3.03(1.82, 5.04)***	1.29(0.77, 2.16)
Alcohol-related illness					
No	1151	4,120,394	2.79	1.00	1.00
Yes	69	43,547	15.8	5.57(4.37, 7.10)***	2.14(1.64, 2.79)***
Anxiety					
No	1171	4,127,221	2.84	1.00	1.00
Yes	49	36,720	13.3	4.62(3.47, 6.15)***	1.38(1.02, 1.87)*
Mental disorders					
No	1158	4,045,473	2.86	1.00	1.00
Yes	62	118,468	5.23	1.73(1.34, 2.24)***	1.21(0.93, 1.59)
Insomnia					
No	1135	4,082,986	2.78	1.00	1.00
Yes	85	80,955	10.5	3.67(2.95, 4.58)***	1.24(0.97, 1.57)

CI, confidence interval; HR, hazard ratio; PY, person-years. ^#^Incidence rate per 10,000 person-years. ^$^Multivariable analysis included age, monthly income, urbanization level, occupation category, and comorbidities of schizophrenia, alcohol-related illness, anxiety, mental disorders, and insomnia. ^†^New Taiwan Dollar (NTD), 1 NTD is equal to 0.03 USD. ^‡^The urbanization level was divided into 4 levels based on the population density of the residential area, where level 1 was the most urbanized and level 4 was the least urbanized. ^&^Other occupation categories included those who were primarily retired, unemployed, and low-income people. *P < .05, **P < .01, ***P < .001.

**Table 3** presents that compared with COPD patients without depression, a significantly high SA risk was observed in COPD patients with depression (aHR, 3.09; 95% CI, 2.78–3.44) as well as in patients aged <50 years (aHR, 9.14; 95% CI, 6.02–13.9), 50–64 years (aHR, 6.94; 95% CI, 4.65–10.4), and >65 years (aHR, 4.61; 95% CI, 3.63–5.85); female (aHR, 6.93; 95% CI, 5.16–9.29) or male patients (aHR, 5.13; 95% CI, 4.06–6.48); patients with monthly incomes of NTD <15,000 (aHR, 4.60; 95% CI, 3.38–6.27), 15,000–19,999 (aHR, 5.99; 95% CI, 4.67–7.68), and >20,000 (aHR, 7.50; 95% CI, 4.52–12.5); patients living in areas with urbanization level 1 (aHR, 7.46; 95% CI, 4.83–11.5), level 2 (aHR, 7.74; 95% CI, 5.64–10.6), level 3 (aHR, 5.01; 95% CI, 3.16–7.95), and level 4 (aHR, 3.91; 95% CI, 2.84–5.38); patients employed as office workers (aHR, 6.86; 95% CI, 4.90–9.59), laborers (aHR, 5.43; 95% CI, 4.19–7.03), and other types of workers (aHR, 4.63; 95% CI, 3.14–6.82); patients without any comorbidity (aHR, 6.49; 95% CI, 5.29–7.96); and patients with any one of the comorbidities (aHR, 4.11; 95% CI, 3.06–5.52) (**Table 3**).

**Table 3:** Comparison of incidence and hazard ratio of suicide attempt stratified based on age, sex, and comorbidities between copd patients with and without depression.

	COPD without depression (N=340,694)	COPD with depression (N=21,009)
	Adjusted HR^$^ (95% CI)	Adjusted HR^$^ (95% CI)
**All**	1.00	3.09(2.78, 3.44)***
**Age, year**		
≤49	1.00	9.14(6.02, 13.9)***
50-64	1.00	6.94(4.65, 10.4)***
65+	1.00	4.61(3.63, 5.85)***
P for interaction		< 0.001
**Sex**		
Female	1.00	6.93(5.16, 9.29)***
Male	1.00	5.13(4.06, 6.48)***
P for interaction		0.04
**Monthly income** ^†^		
<15,000	1.00	4.60(3.38, 6.27)***
15,000−19,999	1.00	5.99(4.67, 7.68)***
≥20,000	1.00	7.50(4.52, 12.5)***
P for interaction		0.02
**Urbanization level** ^‡^		
1 (highest)	1.00	7.46(4.83, 11.5)***
2	1.00	7.74(5.64, 10.6)***
3	1.00	5.01(3.16, 7.95)***
4 (lowest)	1.00	3.91(2.84, 5.38)***
P for interaction		0.002
**Occupation category** ^&^		
Office worker	1.00	6.86(4.9, 9.59)***
Laborer	1.00	5.43(4.19, 7.03)***
Other	1.00	4.63(3.14, 6.82)***
P for interaction		0.03
**Comorbidity** ^§^		
None	1.00	6.49(5.29, 7.96)***
With any one	1.00	4.11(3.06, 5.52)***
P for interaction		0.06

CI, confidence interval; HR, hazard ratio. ^$^Multivariable analysis included age, monthly income, urbanization level, and comorbidities of schizophrenia, alcohol-related illness, anxiety, mental disorders, insomnia, diabetes mellitus, hypertension, hyperlipidemia, chronic obstructive pulmonary disease, coronary artery disease, stroke, and cirrhosis. ^†^New Taiwan Dollar (NTD), 1 NTD is equal to 0.03 USD. ^‡^The urbanization level was divided into 4 levels based on the population density of the residential area, where level 1 was the most urbanized and level 4 was the least urbanized. ^&^Other occupation categories included those who were primarily retired, unemployed, and low-income people. ^§^Individuals with schizophrenia, depression, alcohol-related illness, anxiety, mental disorders, and insomnia were classified into the comorbidity group. ***P < .001.

The event numbers, incidence rates, and HRs of SDO of COPD patients with and without depression are shown in **Table 4**. Patients with COPD (aHR: 3.59; 95% CI, 3.37–3.81), COPD patients without depression (aHR, 3.24; 95% CI, 3.04–3.45), and COPD patients with depression (aHR, 10.0; 95% CI, 8.99–11.2) had a significantly higher SDO risk than controls. Specifically, COPD patients with depression had a higher incidence (71.2 per 10,000 person-years) and risk of SDO than COPD patients without depression (aHR, 3.09; 95% CI, 2.77–3.40) (**Table 4**).

**Table 4:** Overall incidence of suicidal drug overdose (per 10,000 person-years) and estimated hazard ratio according to copd patients with or without depression through the cox method.

	Comparison cohort	Total COPD	COPD without depression	COPD with depression
Variable	(N=109,040)	(N=54,520)	(N=48,879)	(N=5,641)
Person-years				
Event, n	1835	2741	2265	476
Rate^#^	6.11	23.8	20.9	71.2
Crude HR (95% CI)	1(Reference)	3.83(3.61, 4.07)***	3.36(3.16, 3.58)***	11.5(10.4, 12.7)***
Adjusted HR^$^ (95% CI)	1(Reference)	3.59(3.37, 3.81)***	3.24(3.04, 3.45)***	10.0(8.99, 11.2)***
Crude HR (95% CI)			1(Reference)	3.42(3.10, 3.78)***
Adjusted HR^$^ (95% CI)			1(Reference)	3.09(2.77, 3.4)***

^#^Incidence rate per 10,000 person-years. ^$^Multivariable analysis included age, monthly income, urbanization level, and comorbidities of schizophrenia, alcohol-related illness, anxiety, mental disorders, insomnia, diabetes mellitus, hypertension, hyperlipidemia, chronic obstructive pulmonary disease, coronary artery disease, stroke, and cirrhosis. ***P < .001.

Moreover, to evaluate whether patients with COPD and depression might have higher SA and SDO risks than patients with depression without COPD, we further compared COPD patients with depression (n = 19,725) with non-COPD patients with depression who were frequency matched based on sex, age, and index year (**Supplementary Tables**). COPD patients with depression had a moderately high risk of SA (aHR, 3.59; 95% CI, 2.85–4.52) and SDO (aHR, 4.97; 95% CI, 4.20–5.88) than non-COPD patients with depression.

To assess the incidence and risk of different SDO types, patients with SDO were classified based on the different methods of suicide (**Table 5**), such as poisoning through drugs or medical substances, benzodiazepine-based tranquilizers, and other methods. Compared with controls, patients with COPD (aHR, 2.82; 95% CI, 2.44–3.26), COPD patients without depression (aHR, 2.37; 95% CI, 2.04–2.77), and COPD patients with depression (aHR, 10.9; 95% CI, 8.57–13.8) had a significantly high risk of poisoning through drugs or medicinal substances. Specifically, COPD patients with depression had a higher incidence (16.5 per 10,000 person-years) and risk of poisoning through drugs or medical substances (aHR, 4.59; 95% CI, 3.62–5.83) than those without depression.

**Table 5:** Overall incidence with different methods of suicidal drug overdose (per 10 000 person-years) and estimated hazard ratio according to copd patients with or without depression through the cox method.

	Comparison cohort	Total COPD	COPD without depression	COPD with depression
Variable	(N=721,550)	(N=361,703)	(N=340,694)	(N=21,009)
**Poisoning by drugs or medical substances**				
Event, n	366	445	335	110
Rate^#^	1.22	3.87	3.09	16.5
Crude HR (95% CI)	1(Reference)	3.15(2.74, 3.62)***	2.52(2.17, 2.92)***	13.4(10.8, 16.6)***
Adjusted HR^$^ (95% CI)	1(Reference)	2.82(2.44, 3.26)***	2.37(2.04, 2.77)***	10.9(8.57, 13.8)***
Crude HR (95% CI)			1(Reference)	5.34(4.31, 6.63)***
Adjusted HR^$^ (95% CI)			1(Reference)	4.59(3.62, 5.83)***
**Poisoning by benzodiazepine-based tranquilizers**				
Event, n	172	0.57	192	70
Rate^#^	2.62	2.28	1.77	10.5
Crude HR (95% CI)	1(Reference)	3.91(3.22, 4.74)***	3.04(2.47, 3.74)***	18.0(13.7, 23.8)***
Adjusted HR^$^ (95% CI)	1(Reference)	3.34(2.73, 4.08)***	2.76(2.24, 3.41)***	13.9(10.2, 19.0)***
Crude HR (95% CI)			1(Reference)	5.95(4.52, 7.82)***
Adjusted HR^$^ (95% CI)			1(Reference)	4.97(3.66, 6.73)***
**Others**				
Event, n	1297	2034	1738	296
Rate^#^	4.32	17.7	16.0	44.3
Crude HR (95% CI)	1(Reference)	4.01(3.74, 4.31)***	3.64(3.39, 3.91)***	10.1(8.89, 11.4)***
Adjusted HR^$^ (95% CI)	1(Reference)	3.85(3.58, 4.14)***	3.56(3.31, 3.83)***	9.35(8.16, 10.7)***
Crude HR (95% CI)			1(Reference)	2.78(2.45, 3.14)***
Adjusted HR^$^ (95% CI)			1(Reference)	2.59(2.27, 2.95)***

^#^Incidence rate per 10 000 person-years. ^$^Multivariable analysis included age, monthly income, urbanization level, and comorbidities of schizophrenia, alcohol-related illness, anxiety, mental disorders, insomnia, diabetes mellitus, hypertension, hyperlipidemia, chronic obstructive pulmonary disease, coronary artery disease, stroke, and cirrhosis. ***P < .001.

Compared with controls, patients with COPD (aHR, 3.34; 95% CI, 2.73–4.08), COPD patients without depression (aHR, 2.76; 95% CI, 2.24–3.41), and COPD patients with depression (aHR, 13.9; 95% CI, 10.2–19.0) had a significantly high risk of poisoning through benzodiazepine-based tranquilizers. Specifically, COPD patients with depression had a higher incidence (10.5 per 10,000 person-years) and risk of poisoning through benzodiazepine-based tranquilizers (aHR, 4.97; 95% CI, 3.66–6.73) than those without depression.

Compared with controls, a significantly high risk of poisoning through other methods was observed in patients with COPD (aHR, 3.85; 95% CI, 3.58–4.14), COPD patients without depression (aHR, 3.56; 95% CI, 3.31-3.83), and COPD patients with depression (aHR, 9.35; 95% CI, 8.16–10.7). Specifically, a significantly higher risk of poisoning through other methods was observed in COPD patients with depression (aHR, 2.59; 95% CI, 2.27–2.95) than in those without depression (**Table 5**).

## Discussion

Despite the urgency and necessity of reducing suicidality worldwide, several developed countries have not invested sufficient resources in SA research and prevention (5). An obvious imbalance exists between the magnitude of the suicide problem and the knowledge required to address it. The present study involved a 13-year follow-up of patients aged ≥20 years, and the results revealed that 5.81% of patients with COPD developed depression. One of our previous studies with a similar design showed that the incidence of SA and SDO in patients with depression was 3.76 and 6.97, respectively, per 10,000 person-years (15). The current study revealed that the incidence of SA and SDO in COPD patients with depression was 29.7 and 71.2 per 10,000 person-years, respectively. COPD patients with depression had a 13.6- and 3.09-fold higher SA risk than did controls and COPD patients without depression, respectively. Furthermore, COPD patients with depression had a 10.0- and 3.09-fold higher SDO risk than did controls and COPD patients without depression, respectively. Contrary to the belief that COPD moderately increases SA and SDO risks in patients with depression, this large-scale study revealed enhancement of SA and SDO risks in COPD patients with coexisting depression. Furthermore, regardless of the confounding effects of socioeconomic factors such as monthly income, urbanization level, and occupation category, age less than 50 years is a specifically high risk factor for SA in COPD patients that interact with depression. Moreover, no differences were observed in SA risk between male and female patients in this study when considering the overwhelming enhancement effects of depression on COPD.

We usually evaluate suicidality using self-reported passive and active death-related thoughts collected through a questionnaire with a binary (yes/no) response. Using this method, suicidal ideation can be investigated; however, valid methods for assessing suicidal behavior, SA, and suicidal death risk are unavailable. Moreover, in the United Kingdom, 1 of 5 adults considers suicide; however, only one in 15 attempts suicide (6). Another study reported that only a small proportion of individuals who exhibit suicidal ideation actually attempt suicide (16). Our study showed proportionally extreme increases in SA and SDO risks resulting from the effects of comorbid depression on COPD. Compared with the annual SA prevalence of 0.4%–0.5% in Western countries (2, 3), the prevalence in our patients with COPD and depression was lower than expected. However, as a nonviolent method of SA, SDO is frequently noted in Taiwanese patients with COPD and depression. Although more than 95% of the people who attempt SDO could eventually survive in Europe (17), SDO causes considerable burden in Asian countries; hence, SA through SDO should be effectively prevented (18, 19). Our results implied that COPD patients could be dangerously abuse prescribed medicines and tranquilizers for attempting suicide through overdose, especially when these patients have comorbid depression. This study reiterates that medicines and tranquilizers used for self-poisoning are highly accessible in Taiwan, because of the restricted access to firearms in Taiwan, similar to most Asian countries. Therefore, careful and long-term accurate assessment should be performed before prescribing multiple medicines or tranquilizers to COPD patients with depression. Moreover, rather than focusing on their suicidal ideation only, awareness of the relationships of different physical disorders with depression would help identify patients at a high SA risk, thereby preventing suicidal death.

However, depression mediates the relationship between value strain, deprivation strain, aspiration strain, coping strain, and suicidality (8). Psychological strain may cause suicidal behaviors or impulsive suicide in patients with a lack of social support/coping capacities (20), poor functional status (21, 22), and disorders of subjective perception including anxiety, panic, or other mental disorders (23, 24). Those might partially explain the enhancement of depression on suicidal behaviors in COPD patients. Nonetheless, the actual interactions between COPD and depression on suicidality warrant additional investigational studies. Given the neuro-psychological pathology of COPD, considerable biological evidence has demonstrated that chronic inflammation, such as that in COPD, may increase the pathologies over the blood–brain barrier, glutamate regulation, microglia activation, and autoimmune response (25, 26). These can affect patients’ physical endurance, sleep, chronic cognition, and associated psychosocial sequelae, thereby contributing to patients’ suicidality. Furthermore, oxidative stress in COPD could decrease serotonin synthesis owing to the effects of hypoxemia on tryptophan hydroxylase, and oxidative stress could cause depression and suicidality (27). These findings revealed a notably bidirectional relationship between depression and COPD that increases suicidality in affected patients.

Our findings proved that depression is an extreme factor enhancing suicidal behaviors in patients with COPD. However, this study has some limitations. First, this was a retrospective study that used NHIRD claims data with hospitalization records related to COPD and depression during the study period. Although the accuracy of inpatient data is assured because of severe penalty associated with providing incorrect data, identifying SA, SDO, COPD, and depression based on the ICD-9-CM coding system is still associated with a possibility of underdiagnosis and underestimation. Minor SA and SDO in patients with COPD might not require medical services, resulting in the underestimation of SA and SDO risks. Second, we could not directly contact patients to determine the severity of COPD and depression, their actual taken medications, or treatments for their disorders because patients’ identities were anonymized in the NHIRD. These details may confound their suicidality. Finally, even though our study design controlled for numerous confounders to the best of our knowledge, some unmeasured or unknown confounders may remain. Further large-scale studies are necessary to gain a comprehensive understanding of suicidality in patients with COPD with and without depression; such studies would help establish an effective prevention system in Taiwan and worldwide.

## Conclusions

COPD and depression increase the risk of suicidality individually and synergistically. COPD patients with depression have increased SA and SDO risks. Specifically, COPD patients with depression aged less than 50 years have high SA risk. Our findings provide crucial information for suicide prevention for clinicians and the governments of Taiwan and other Asian countries. In COPD patients with depression, long-term medicines or tranquilizers should be prescribed and dispensed with caution.

## Data Availability Statement

The datasets generated for this study are available on request to the corresponding authors.

## Ethics Statement

The studies involving human participants were reviewed and approved by the research Ethics Committee of China Medical University and Hospital in Taiwan approved the study (CMUH104-REC2-115-CR4). Written informed consent for participation was not required for this study in accordance with the national legislation and the institutional requirements.

## Author Contributions

Study conception/design: TH, C-HK. Provision of study material and patients: C-HK. Collection and assembly of data, data analysis and interpretation, manuscript writing, and final approval of manuscript: all authors.

## Conflict of Interest

The authors declare that the research was conducted in the absence of any commercial or financial relationships that could be construed as a potential conflict of interest.

## Abbreviations

aHR, adjusted hazard ratio; CI, confidence interval; COPD, chronic obstructive pulmonary disease; NHI, National Health Insurance; NHIRD, National Health Insurance Research Database; NTD, New Taiwan Dollar; HR, hazard ratio; ICD-9-CM, International Classification of Diseases, Ninth Revision, Clinical Modification; SA, suicide attempt; SDO, suicidal drug overdose.
