# Supplementary material for: Suicide Attempt and Suicidal Drug Overdose in Chronic Obstructive Pulmonary Disease Patients With or Without Depression
Source: Front Psychiatry. 2020 Apr 15;11:270. doi: 10.3389/fpsyt.2020.00270 (PMC7174688; doi:10.3389/fpsyt.2020.00270)
Supplement: Supplementary file 1 [file Table_1.docx]

| **Supplementary Table 1.** Distribution of Demographic Characteristics and Comorbidities Among COPD With Depression and Non-COPD With Depression Cohorts | | | | | |
| --- | --- | --- | --- | --- | --- |
|  | Non-COPD with  depression (N=19,725) | | COPD with depression (N=19,725) | |  |
|  | n | % | n | % | p-value^a^ |
| **Sex** |  |  |  |  | 0.99 |
| Women | 7779 | 39.4 | 7779 | 39.4 |  |
| Men | 11946 | 60.6 | 11946 | 60.6 |  |
| **Age stratified** |  |  |  |  | 0.90 |
| ≤ 49 | 2094 | 10.6 | 2066 | 10.5 |  |
| 50-64 | 3671 | 18.6 | 3671 | 18.6 |  |
| ≥ 65 | 13960 | 70.8 | 13988 | 70.9 |  |
| Age, mean ± SD^§^ | 67.8±12.8 | | 70.4±14.1 | | <0.001 |
| **Monthly income**^†^ |  |  |  |  | <0.001 |
| < 15,000 | 7318 | 37.1 | 9108 | 46.2 |  |
| 15,000−19,999 | 8216 | 41.7 | 8299 | 42.1 |  |
| ≥ 20,000 | 4191 | 21.3 | 2318 | 11.8 |  |
| **Urbanization level**^‡^ |  |  |  |  | <0.001 |
| 1 (highest) | 5394 | 27.4 | 4039 | 20.5 |  |
| 2 | 5697 | 28.9 | 5813 | 29.5 |  |
| 3 | 2916 | 14.8 | 3003 | 15.2 |  |
| 4 (lowest) | 5718 | 28.9 | 6870 | 34.8 |  |
| **Occupation category**^＆^ |  |  |  |  | <0.001 |
| Office worker | 8380 | 42.5 | 6002 | 30.4 |  |
| Laborer | 7800 | 39.5 | 7980 | 40.5 |  |
| Other | 3545 | 18.0 | 5743 | 29.1 |  |
| **Comorbidity** |  |  |  |  |  |
| Schizophrenic | 524 | 2.66 | 463 | 2.35 | 0.049 |
| Alcohol-related illness | 808 | 4.10 | 1534 | 7.78 | <0.001 |
| Anxiety | 1012 | 5.13 | 2158 | 10.9 | <0.001 |
| Sleep disorders | 1559 | 7.90 | 3768 | 19.1 | <0.001 |
| ^a^Chi-square test; ^§^NOVA. ^†^New Taiwan Dollar (NTD), 1 NTD is equal to 0.03 USD. ^‡^The urbanization level was divided into 4 levels based on the population density of the residential area; level 1 was the most urbanized and level 4 was the least urbanized. ^＆^Other occupation categories included those who were primarily retired, unemployed, and low-income people. | | | | | |

| **Supplementary Table 2.** Overall Incidence of Suicide Attempt and Suicidal Drug Overdose (per 10 000 person-y) and Estimated Hazard Ratio According to COPD Patients With Depression or Non-COPD with Depression Through the Cox Method | | |
| --- | --- | --- |
|  | Non-COPD with Depression | COPD Patients With Depression |
| Variable | (N=19,725) | (N=19,725) |
| Suicide Attempt |  |  |
| Person-years | 172269 | 67647 |
| Event, n | 131 | 201 |
| Rate^#^ | 7.60 | 29.7 |
| Crude HR (95% CI) | 1(Reference) | 3.72(2.98, 4.65)*** |
| Adjusted HR^$^ (95% CI) | 1(Reference) | 3.59(2.85, 4.52)*** |
| Suicidal Drug Overdose |  |  |
| Person-years | 172052 | 66847 |
| Event, n | 219 | 476 |
| Rate^#^ | 12.7 | 71.2 |
| Crude HR (95% CI) | 1(Reference) | 5.34(4.55, 6.28)*** |
| Adjusted HR^$^ (95% CI) | 1(Reference) | 4.97(4.20, 5.88)*** |
| ^#^Incidence rate per 10 000 person-years. ^$^Multivariable analysis included age, monthly income, urbanization level, and comorbidities of schizophrenia, alcohol-related illness, anxiety, insomnia, diabetes mellitus, hypertension, hyperlipidemia, chronic obstructive pulmonary disease, coronary artery disease, stroke, and cirrhosis. ****P* < .001. | | |
